# The effect of lipoaspirates vs. dissected abdominal fat on breast cancer cells in vitro

**DOI:** 10.1186/s40001-017-0251-3

**Published:** 2017-03-21

**Authors:** Faris Almarzouqi, Hans-Oliver Rennekampff, Jan-Philipp Stromps, Ziyad Alharbi, Norbert Pallua

**Affiliations:** Department of Plastic Surgery, Hand Surgery and Burn Center, RWTH University Hospital, Pauwelsstrasse 30, 52074 Aachen, Germany

**Keywords:** Fat grafting, Breast cancer, Breast reconstruction

## Abstract

**Background:**

Cancer cells are typically surrounded by stromal cells and embedded in extracellular matrix (ECM). The stromal compartment interacts with cancer cells to promote growth and metastasis. For decades, autologous fasciocutaneous flaps have been safely applied for breast reconstruction after mastectomy. In contrast, the safety of fat grafting (lipofilling) procedure has been under debate regarding the risk of cancer recurrence.

**Methods:**

Harvested fat tissue (lipoaspirates) and dissected abdominal fat (DAF) were co-cultured with MCF-7 breast cancer cells. The vitality of MCF-7 cells was measured using AlamarBlue^®^ consecutively for 5 days. ECM degradation was determined by detection of matrix metalloproteinase-1 (MMP-1) expression in MCF-7 cells. Integrin α2 was measured by Western blot to assess the degree of adhesion and motility of MFC-7 cells.

**Results:**

The MCF-7 proliferation increased substantially when co-cultured with fat tissue. However, there was no significant difference between the proliferation stimulating effects of lipoaspirates and DAF. Similarly, MMP-1 protein expression was equally elevated in MCF-7 cells by both lipoaspirates and DAF. Importantly, MCF-7 cells showed an increased level of integrin α2 once co-cultured with either lipoaspirates or DAF.

**Conclusion:**

Fat tissue increases the proliferation of MCF-7 cells in vitro. Our data suggest that lipoaspirates as well as DAF might possess a considerable potency to promote tumorigenic growth of breast cancer cells. Thus, clinical trials are needed to address the safety of lipofilling by breast reconstruction surgery after mastectomy.

## Background

Cancer cells are typically surrounded by stromal cells like mesenchymal, inflammatory and vascular cells. Stromal cells support cancer cells by providing mediators for cell proliferation, inflammation and angiogenesis [1]. In addition, cancer cells are embedded in extracellular matrix (ECM), which plays crucial roles in modulation of stromal compartment and cancer cell invasion. Modulation of the stromal compartment is exerted by matrix metalloproteinases (MMPs) [2]. MMPs are involved in many physiological and pathological processes in the human body, which are normally present in tissues at low levels and increase dramatically during any changes [3, 4]. Overexpression and activation of MMPs have been implicated in pathological cleavage of connective tissue by arthritis [5], cardiovascular disease [6], and tumor progression [7]. However, the process of metastases is complex. In order to migrate, cell adhesion and motility molecules are required, after cleavage of the connective tissue, to interact with ECM as well as vascular endothelium to the secondary site [8, 9]. Integrin is a family of transmembrane receptors consist of α and β subunits and serves as cell–cell as well as cell–matrix adhesion molecule [10]. In normal and tumor cells, integrins have a central function in intracellular signaling pathways that lead to cell proliferation, differentiation and migration [11].

After initial breast cancer surgery like mastectomy or breast conserving surgery and subsequent oncological therapy, up to 95.5% of these women are considered for breast reconstruction including prosthetic implants and flaps [12]. The latter represents a wide variety of options such as myocutanoeus flaps, e.g., the latissimus dorsi [13] as a regional flap, and muscle-sparing Transvers Rectus Abdominis Myocutaneous flap “msTRAM” as a free tissue transfer [14]. The Deep Inferior Epigastric artery Perforator flap ‘DIEP’ from the abdominal wall has also been successfully applied for breast reconstruction [15]. DIEP flaps provide a homogenous rounded breast and are currently considered as a safe procedure in the oncological aspect and referred to as the “gold standard” by many authors [15, 16].

Since the initial report [17] in 1987, autologous grafting of suspended adipose tissue (lipofilling) has become increasingly popular for cosmetic purposes and breast reconstruction worldwide. Concurrently, the American Society of Plastic Surgeons (ASPS) criticized this method of fat grafting that can compromise breast cancer detection [18]. Nowadays, many plastic surgeons question the safety of the procedure as well, discuss the amount of fat tissue reabsorption and doubt the screening results because of micro-calcification [19]. Ultimately, it remains unknown whether autologous fat grafting enhances the breast cancer recurrence or even initiates it at the first place. Adipose tissue and importantly lipoaspirates is a rich source of growth factors, like basic fibroblast growth factor (bFGF), insulin-like growth factor 1 (IGF-1), vascular endothelial growth factor (VEGF), and platelet-derived growth factor (PDGF-BB) [20]. Besides, mature adipocytes are able to produce considerable amounts of estrogen via aromatase-mediated conversion of androgen to estrogen. All these factors are able to potentiate the growth of breast carcinoma cells through a paracrine mechanism [21–24]. Indeed, obesity and weight gain in adult females correlate with an increasing risk of breast cancer [25]. Yet, it remains unclear whether dissected abdominal fat 'en bloc' (DAF) and suspended adipose cells like lipoaspirates differ in their abilities to stimulate tumor growth and invasion.

Within this study, we observed and compared the effects of harvested fat (lipoaspirates) and DAF on breast cancer cells in vitro. We measured the proliferation of the human breast cancer cells line MCF-7 co-cultured with lipoaspirates or DAF. MMP-1 and integrin α2 expression of tumor cells were also detected in both circumstances.

## Methods

All surgical procedures took place in the Department of Plastic Surgery, Hand Surgery, and Burn Center at RWTH University Hospital, Aachen, Germany. Each patient signed the consent form. The protocol and the use of human materials were approved by the ethics committee of the Faculty of Medicine at RWTH University in Aachen, Germany (Name in German: Ethik-Kommission des Universitätsklinikums Aachen, Votum Number: EK206/13). The experiments were carried out in compliance with Declaration of Helsinki in its current form.

### Study design

**Fig. 1:**
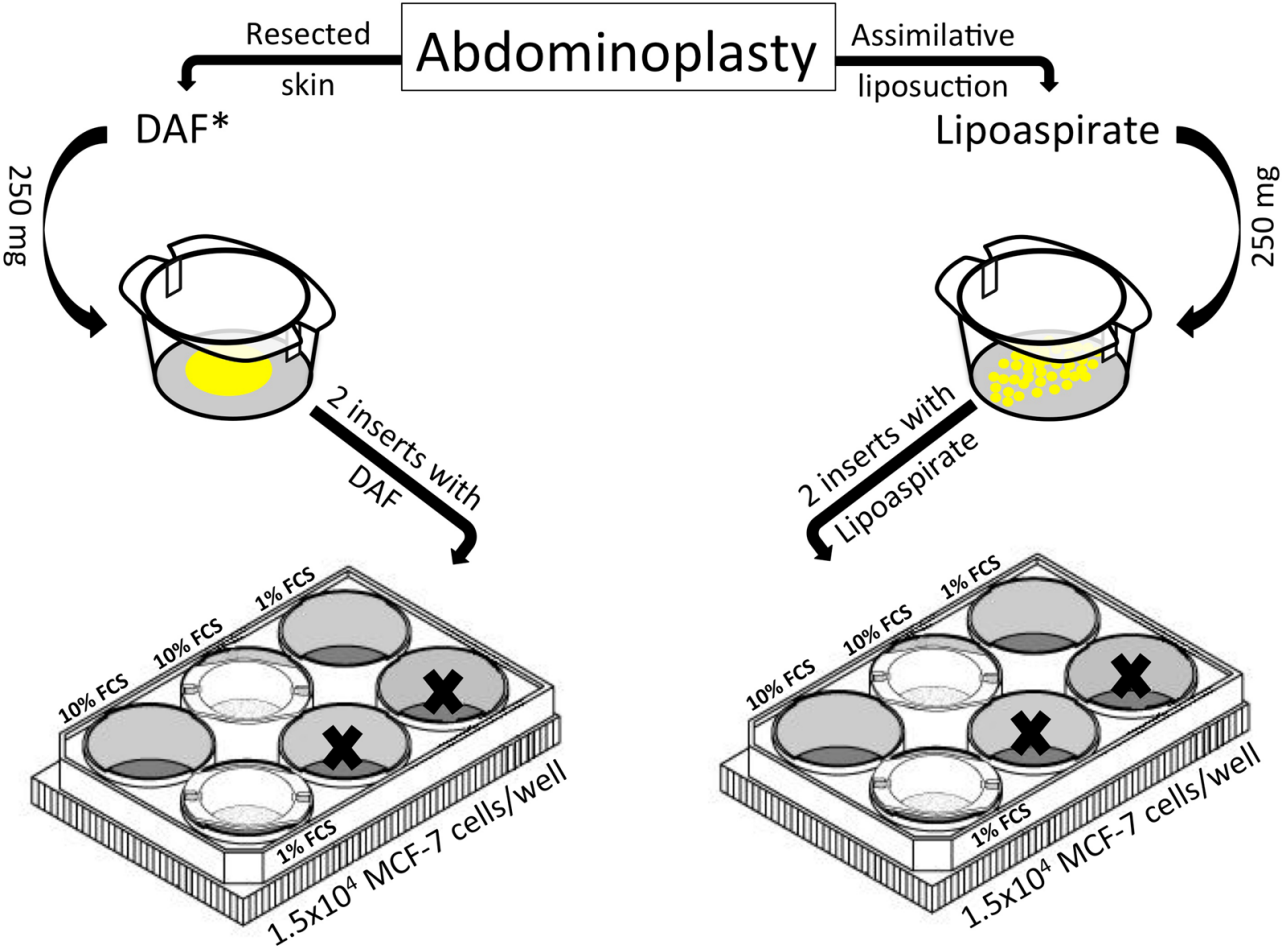
The result after Abdominoplasty and liposuction is a subcutaneous fat from the resected skin flap *left* and lipoaspirates from liposuction *right*. Each fat sample was adjusted to 250 mg and placed into an insert with a membrane. The inserts were placed in 6-well plate containing 1.5 × 10^4^ adherent cancer cells. 2-well were used for a medium with 10% FCS and other 2-well for 1% FCS. The last 2-well was left unused

We selected 8 healthy female patients (*n* = 8) who underwent elective abdominoplasty and liposuction. The ages of the patients were between 25 and 64 years (mean = 46.75 years). The Body Mass Index (BMI) was between 24 and 45 (mean = 32). All lipoaspirates included in this study have been harvested only from the abdominal wall, and each sample was processed as described by Coleman [26]. The lipoaspirates was divided into 250 mg samples. Simultaneously, 250 mg fat tissue (en bloc) from the same patient was dissected from abdominal fat (DAF). Each lipoaspirates or DAF sample was placed into an insert with a 0.4 µm pore size membrane [Thermo Fisher Scientific Inc., USA]. The inserts were placed in 6-well plate containing 1.5 × 10^4^ adherent MCF-7 breast cancer cells per well. Control wells were cultured without addition of fat tissue. Two sets of parallel experiments were performed using 1 or 10% fetal calf serum (FCS) (Fig. 1).

### Cell culture

The human breast cancer cell line MCF-7 was obtained from the American Type Culture Collection [HTB 22; derived from a breast adenocarcinoma of 69-year-old Caucasian woman]. MCF-7 cells were maintained in RPMI 1640 supplemented with 5 ml of Minimal Essential Medium non-essential amino acids (NEAA) [PAA, Linz, Austria], 1 mM sodium pyruvate [PAA, Linz, Austria], 1% penicillin and 1% streptomycin [PAA, Linz, Austria], 10 µg/ml human insulin [Biochrom AG, Berlin, German], and 1 or 10% FCS [PAA, Linz, Austria]. Cells were cultured at 37 °C in a humidified atmosphere of 5% CO_2_.

### Proliferation assay

Proliferation of MCF-7 cells was daily monitored using AlamarBlue^®^ dye [Life technologies]. This dye is a redox indicator that yields a colorimetric change and a fluorescent signal in response to metabolic activity of cells. The inserts holding fat tissue samples were removed before the assay and kept in pre-warmed medium. The MCF-7 culture medium was adjusted to 1 ml in each well and 100 µl of the AlamarBlue^®^ reagent was added to each well. MCF-7 cell were incubated at 37 °C and 5% CO_2_ for 2 h and subjected to fluorescence measurement. After data collection, the reagent was replaced by fresh medium and the inserts with fat tissue samples were placed back to the corresponding well of MCF-7 cells. After 5 days, cells were harvested and all samples (medium, fat tissue and cell lysates) were kept by −80 °C for further analysis.

### Elisa

Matrix metalloproteinase-1 expression in MCF-7 cells was measured using an ELISA kit according to the manufacturer’s instructions and in two parallel independent experiments [Human Total MMP-1 DuoSet “DY901”, R&D Systems, Minneapolis, MN, USA]. The obtained data were normalized to the total protein concentration. Mean value of the two independent experiments is presented in corresponding figures in this study.

### Western blot

Integrin α2 expression in MCF-7 cells was detected by Anti-Integrin α2 antibodies using Western blot according to the manufacturer’s instructions and in two parallel independent experiments [Anti-Integrin alpha 2 antibody (EPR5789), Abcam plc, Cambridge, UK]. Blots were detected with the LAS-3000 image analyzer (Fujifilm) and protein bands were quantified densitometrically using AIDA software (raytest). Relative protein levels were obtained by normalization to Tubulin. Mean value of the two independent experiments is presented in corresponding figures in this study.

### Statistical analysis

Values are expressed as the arithmetic mean ± standard error of mean (SEM). All assays were performed in duplicate at two different times. The statistical significance of differences between groups in the proliferation assay was determined via linear regression, whereas significance of MMP-1 and Integrin α2 expression was measured by *t* test. The differences were considered statistically significant when *p* < 0.05. All the analyses were performed using Prism^®^ 5 for Macintosh [GraphPad Software Inc., La Jolla, CA].

## Results

### Proliferation of MCF-7

**Fig. 2:**
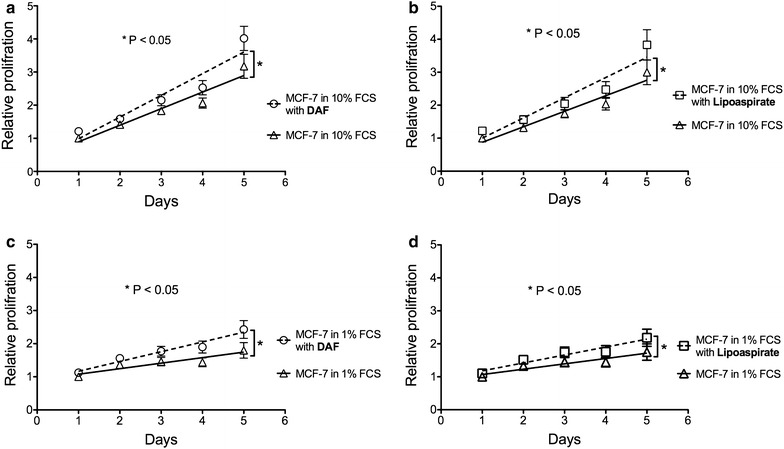
**a** Demonstrates a significant increase of the relative proliferation of MCF-7 co-cultured with DAF comparing to MCF-7 cells alone (control group) in RPMI 1640 medium with 10% FCS; and in 1% FCS (**b**); **c**, **d** Show the difference in relative proliferation between MCF-7 co-cultured with lipoaspirates and control MCF-7 in 10 and 1% FCS consequently. *Linear regression*

**Fig. 3:**
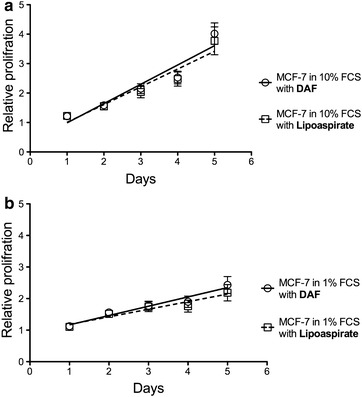
**a** Demonstrates no statistically difference in the relative proliferation between MCF-7 co-cultured with DAF and lipoaspirates in 10% FCS; and **b** in 1% FCS. *Linear regression*

To determine the effect of co-cultured fat tissue on the proliferation of breast cancer cells, we harvested fat tissue from various patients by two different techniques, liposuction or dissection from the skin flap. MCF-7 breast cancer cells were cultured with the equal amounts of harvested fat tissue or without as control. We used AlamarBlue^®^ assay to quantitatively monitor the proliferation of MCF-7 cells. The relative proliferation rates of MCF-7 cells were determined by comparison of their metabolic activity with or without supplemented fat tissue every 24 h for 5 consecutive days. The data are summarized in Fig. 2a–d and show a significant increase in viability of MCF-7 cells when co-cultured with lipoaspirates or DAF compared to the controls lacking fat tissue. Most importantly, there is no significant difference between the effects of DAF and lipoaspirates on relative MCF-7 proliferation rates (Fig. 3). This has been confirmed for 8 different female patients aged between 25 and 64 year old and with a BMI between 24 and 45.

### Expression of MMP-1

**Fig. 4:**
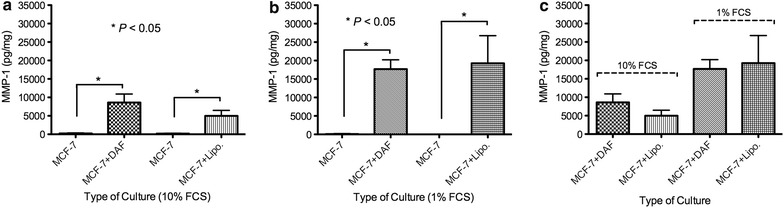
ELISA levels of MMP-1 in MCF-7 lysates after 5 days of co-cultivation: **a** MCF-7 co-cultured with DAF as well as lipoaspirates in RPMI 1640 medium with 10% FCS shows marked increase in MMP-1 levels; **b** MCF-7 co-cultured with DAF as well as lipoaspirates in RPMI 1640 medium with 1% FCS; **c** comparison between co-cultured MCF-7 with DAF and lipoaspirates in 10 and 1% FCS reveal no significance. * T* test

Cell invasion is essential in metastasis of tumors. It involves migration of cancer cells through an extracellular matrix (EMC) or basement membrane extract (BME) by enzymatic degradation of these barriers. Matrix metalloproteinase-1 (MMP-1) is a crucial proteinase that regulates cell invasion directly by modeling and remodeling of the extracellular matrix. We measured MMP-1 protein expression using a direct ELISA test to evaluate the invasion potential of MCF-7 cells with or without co-culture of fat tissue (lipoaspirates or DAF). MCF-7 cells are generally recognized as a non-invasive cell line. Thus, our data show no or undetectable level of MMP-1 protein in total extract from MCF-7 cells. Co-cultivation of fat tissue either lipoaspirates or DAF express a significant increase of MMP-1 levels (Fig. 4a, b). In the matter of comparison between lipoaspirates and DAF co-cultured cancer cells, we found no statistical difference (Fig. 4c). However, in hunger (1% FCS) medium, we noticed higher existence of MMP-1 comparing to normally saturated (10% FCS) medium.

### Expression of integrin α2

**Fig. 5:**
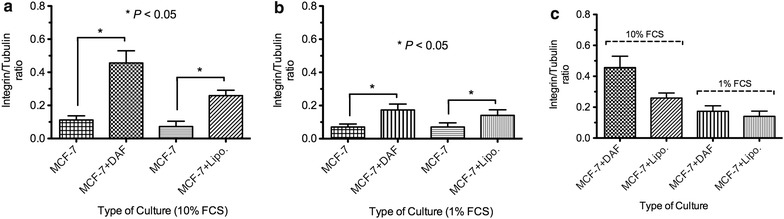
Densitometrically quantified relative Integrin α2 levels after normalizion to tubulin in MCF-7 Lysates after 5 days of co-cultivation: **a** levels of Integrin α2/tubulin are raised in co-cultured MCF-7 with DAF as well as lipoaspirates in RPMI 1640 medium with 10% FCS; and with 1% FCS (**b**); **c** comparison between co-cultured MCF-7 with DAF and lipoaspirates in 10 and 1% FCS revealed no significance.* T* test

**Fig. 6:**
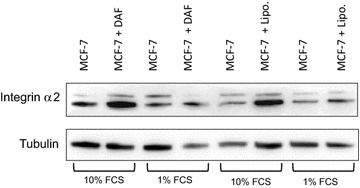
Western blot analysis confirming the expressions of Integrin alpha2 proteins, and Tubulin proteins in MCF-7 cells, with and without combination of DAF or lipoaspirates. Western blotting was performed using the anti-integrin alpha2 antibody. The molecular weights are shown on the *left*

We measured the Integrin α2 to assess the ability of MCF-7 cells to bind and interact with the surrounding environment in order to migrate. Co-cultured MCF-7 cells expressed higher level of Integrin α2 regardless to the type of fat tissue (DAF or lipoaspirates) than control groups (Figs. 5 and 6). This finding has been observed in medium, 10 and 1% FCS; however, in latter medium, the Integrin α2 was expressed in lower levels compared to the former one.

## Discussion

Major achievements have been made in breast reconstruction following mastectomy surgery. The standard of care to reconstruct the breast has focused on tissue expander/implants and the use of flaps [27]. Although often very effective in restoring the contour of the breast, both procedures are not without complications. Donor site morbidity is a major issue in flap procedures [28]. Capsule formation after prosthetic implants and radiotherapy-induced complications are still challenging [29, 30]. In general, autologous methods are favorable in terms of long-term stability of results. However, patient safety must have priority in choosing the method of reconstruction.

Fat grafting has become frequently used for breast reconstruction after mastectomy [31]. Petit et al. concluded in a multicenter study that lipofilling of the breast is a safe procedure after most oncological breast surgeries [32]. In contrast, Yu et al. demonstrated in an animal model, that co-injection of adipocytes-derived stem cells (ASCs) promote proliferation of tumor cells [33]. Furthermore, several other studies described the effect of stromal cells on tumorigenesis in terms of cancer survival, proliferation, invasion, and migration [34]. In this study, we report that lipoaspirates significantly increase proliferation of human MCF-7 breast cancer cells in vitro. It is well known that adipocytes contain a variety of tumor promoting factors, even in estrogen-negative cells, like adiponectin, leptin, collagen IV, and hepatocytes growth factor [35–38]. Therefore, not only ASCs enhance tumor growth but also adipocytes as contained in lipoaspirates may stimulate tumor growth. Tumor invasion is a hallmark of malignant tumors. The ability of tumor cells to degrade the ECM indicates the invasion potential of these cells and subsequently migration and metastasis [7, 39]. We detected a significant increase in MMP-1 expression in MCF-7 cells when co-cultured with lipoaspirates. In comparison, MMP-1 was not detectable in control groups (non-co-cultured). Interestingly, MMP-1 showed higher existence in hunger medium (1% FCS) than in saturated medium (10% FCS), which may suggest the higher tendency to invade the ECM under the former medium. Tumor metastasis to secondary sites demands cell motility as well as interactions between tumor cells and the microenvironment. As binding molecule, Integrin α2β1 has shown high expression in tumor cells isolated from secondary sites [40–43]. In our study, the expression of Integrin α2 in Western blot was significantly higher in lipoaspirates-co-cultured MCF-7 cells than the control groups. Riikonen et al. reported an association between Integrin α2β1 and regulation of MMP-1 expression [44]. In their findings, the increase of Integrin α2 levels was positively associated with an elevation of MMP-1 expression. In our study, we could also demonstrate an increase in Integrin α2 levels accompanied with upregulated MMP-1 expression. However, this effect was differentially regulated with higher Integrin α2 levels resulting in only moderate increase of MMP-1 expression over controls. The exact role of different levels of Integrin α2 overexpression is still controversial [45–47].

Apart from lipofilling, DIEP flap is considered as the gold standard procedure to reconstruct the breast especially in Europe [48]. DIEP flap contains fat cells in their regular texture and tissue composition. Our study demonstrates for the first time the impact of adipose tissue ‘en bloc’ as a small subunit of the DIEP flap on breast cancer cells. We have reported a significant increase in proliferation of DAF-co-cultured MCF-7 cells than non-co-cultured cells. Interestingly, it showed statistically no difference between the proliferation of MCF-7 cells co-cultured with DAF or lipoaspirates. Furthermore, the level of MMP-1 in MCF-7 cells was equally elevated by addition of DAF or lipoaspirates. MMP-1 demonstrates not only the invasive activity of cells through degradation of the ECM, but also has a crucial role in the process of metastasis through intercellular signaling [39]. Moreover, the expression of Integrin α2 was significantly increased in DAF-co-cultured MCF-7 cells but with no difference to cells co-cultured with lipoaspirates. This indicates the stimulation of the carcinogenic growth and migration potential of MCF-7 cells by exposure to either forms of fat tissue. However, we cannot rule out that MCF-7 cells in co-culture with fat tissue may induce an increase of MMP-1 expression in the fat tissue itself. Gao and Bing reported that MMP-1 is expressed and secreted by preadipocytes and to a much lesser extent in mature adipocytes, but can be stimulated by macrophage-conditioned medium in both cells types [49].

Proliferation, invasion, and migration are all different pathological behaviors of cancer cells. Ultimately, DAF and lipoaspirates exhibit an equal enhancement of these behaviors on breast cancer cells (MCF-7). It is known that stromal vascular fraction and mature adipocytes share about 60% of their proteome [50]. Therefore, we could understand the tumorigenic effect of DAF and lipoaspirates as well. Before identification of ASCs, obesity was linked to breast cancer in postmenopausal women. Both, the risk and the rate of mortality by breast cancer have been associated with obesity in several studies [51–55]. Over weight has been shown to affect the prognosis through enhancing the metastasis rate and resistance to drugs [56, 57]. Moreover, abdominal obesity and breast cancer were strongly linked in several studies [58, 59]. While ASCs are not the only player in stimulation of tumorigenic growth, the presence of adipocytes have also increased the carcinogenicity as contained in a flap or suspended in lipoaspirates.

## Conclusion

Approximately 80–100% of breast cancer recurrences occur at the tumor bed in the first 3–5 years after initial treatment. Thus, local residing cancer cells and the elimination of recurrence risk will remain the main concerns in reconstructive surgery after mastectomy. Our study addresses the tumorigenic growth of human breast cancer cells by fat tissue transfer in vitro. Independent of harvesting procedure, fat tissue obviously bears the potency to enhance tumorigenic growth activity of cancer cells in vitro. Notably, autologous flaps have been applied without remarkable oncologic complications for many years in vivo. It remains to be investigated whether lipofilling triggers any oncologic diseases in healthy patients. Therefore it seems warranted to assess the cancer recurrence risks and the safety of reconstructive surgery in extended clinical studies in the future.

## Abbreviations

ASPS: American Society of Plastic Surgeons
ASCs: adipose-derived Stem cells
bFGF: basic fibroblast growth factor
BME: basement membrane extract
BMI: body mass index
DAF: dissected abdominal fat
DIEP flap: deep inferior epigastric artery perforator flap
ECM: extracellular matrix
FCS: fetal calf serum
IGF-1: insulin-like growth factor 1
MMPs: matrix metalloproteinases
msTRAM flap: muscle-sparing Transvers Rectus Abdominis Myocutaneous flap
NEAA: non-essential amino acids
PDGF-BB: platelet-derived growth factor
RPMI 1640 medium: Roswell Park Memorial Institute medium
SEM: standard error of mean
VEGF: vascular endothelial growth factor

## References

[1] Egeblad M, Nakasone ES, Werb Z (2010). Tumors as organs: complex tissues that interface with the entire organism. Dev Cell.

[2] Coussens LM, Werb Z (1996). Matrix metalloproteinases and the development of cancer. Chem Biol.

[3] Krześniak-Wszoła N, Bielecki K (2006). Matrix metalloproteinases. Part I. Pol Merkur Lek Organ Pol Tow Lek..

[4] Krześniak-Wszoła N, Bielecki K (2006). Extracellular matrix metalloproteinases. Part II. Pol Merkur Lek Organ Pol Tow Lek..

[5] Hardingham T (2008). Extracellular matrix and pathogenic mechanisms in osteoarthritis. Curr Rheumatol Rep.

[6] Dormán G, Kocsis-Szommer K, Spadoni C, Ferdinandy P (2007). MMP inhibitors in cardiac diseases: an update. Recent Pat Cardiovasc Drug Discov..

[7] Konstantinopoulos PA, Karamouzis MV, Papatsoris AG, Papavassiliou AG (2008). Matrix metalloproteinase inhibitors as anticancer agents. Int J Biochem Cell Biol.

[8] Zetter BR (1990). The cellular basis of site-specific tumor metastasis. N Engl J Med.

[9] Gay LJ, Felding-Habermann B (2011). Contribution of platelets to tumour metastasis. Nat Rev Cancer.

[10] Hynes RO (2002). Integrins: bidirectional, allosteric signaling machines. Cell.

[11] Varner JA, Cheresh DA (1996). Integrins and cancer. Curr Opin Cell Biol.

[12] Spyrou GE, Titley OG, Cerqueiro J, Fatah MF (1998). A survey of general surgeons’ attitudes towards breast reconstruction after mastectomy. Ann R Coll Surg Engl.

[13] Olivari N (1976). The latissimus flap. Br J Plast Surg.

[14] Grotting JC, Urist MM, Maddox WA, Vasconez LO (1989). Conventional TRAM flap versus free microsurgical TRAM flap for immediate breast reconstruction. Plast Reconstr Surg.

[15] Allen RJ, Treece P (1994). Deep inferior epigastric perforator flap for breast reconstruction. Ann Plast Surg.

[16] Gill PS, Hunt JP, Guerra AB, Dellacroce FJ, Sullivan SK, Boraski J (2004). A 10-year retrospective review of 758 DIEP flaps for breast reconstruction. Plast Reconstr Surg.

[17] Bircoll M (1987). Cosmetic breast augmentation utilizing autologous fat and liposuction techniques. Plast Reconstr Surg.

[18] The American Society of Plastic and Reconstructive Surgical Nurses. Report on autologous fat transplantation. ASPRS Ad-Hoc Committee on New Procedures, September 30, 1987. Plast Surg Nurs Off J Am Soc Plast Reconstr Surg Nurses. 1987;7(4):140–1. 3438355

[19] Gutowski KA (2009). Current applications and safety of autologous fat grafts: a report of the ASPS fat graft task force. Plast Reconstr Surg.

[20] Pallua N, Pulsfort AK, Suschek C, Wolter TP (2009). Content of the growth factors bFGF, IGF-1, VEGF, and PDGF-BB in freshly harvested lipoaspirate after centrifugation and incubation. Plast Reconstr Surg.

[21] Mohamed-Ali V, Pinkney JH, Coppack SW (1998). Adipose tissue as an endocrine and paracrine organ. Int J Obes Relat Metab Disord J Int Assoc Study Obes..

[22] Van Landeghem AA, Poortman J, Nabuurs M, Thijssen JH (1985). Endogenous concentration and subcellular distribution of estrogens in normal and malignant human breast tissue. Cancer Res.

[23] Miller WR, Mullen P, Telford J, Dixon JM (1998). Clinical importance of intratumoral aromatase. Breast Cancer Res Treat.

[24] Laharrague P, Fontanilles AM, Tkaczuk J, Corberand JX, Pénicaud L, Casteilla L (2000). Inflammatory/haematopoietic cytokine production by human bone marrow adipocytes. Eur Cytokine Netw.

[25] Huang Z, Hankinson SE, Colditz GA, Stampfer MJ, Hunter DJ, Manson JE (1997). Dual effects of weight and weight gain on breast cancer risk. JAMA, J Am Med Assoc.

[26] Coleman SR (2001). Structural fat grafts: the ideal filler?. Clin Plast Surg.

[27] Piasecki JH, Gutowski KA (2006). Breast reconstruction. Clin Obstet Gynecol.

[28] Lu SM, Nelson JA, Fischer JP, Fosnot J, Goldstein J, Selber JC (2014). The impact of complications on function, health, and satisfaction following abdominally based autologous breast reconstruction: a prospective evaluation. J Plast Reconstr Aesthetic Surg.

[29] Adams WP (2009). Capsular contracture: what is it? What causes it? How can it be prevented and managed?. Clin Plast Surg.

[30] Lentz R, Ng R, Higgins SA, Fusi S, Matthew M, Kwei SL (2013). Radiation therapy and expander-implant breast reconstruction: an analysis of timing and comparison of complications. Ann Plast Surg.

[31] Cordeiro PG (2011). Discussion: the oncologic outcome and immediate surgical complications of lipofilling in breast cancer patients: a multicenter study–Milan-Paris-Lyon experience of 646 lipofilling procedures. Plast Reconstr Surg.

[32] Petit JY, Botteri E, Lohsiriwat V, Rietjens M, De Lorenzi F, Garusi C (2012). Locoregional recurrence risk after lipofilling in breast cancer patients. Ann Oncol Off J Eur Soc Med Oncol ESMO..

[33] Yu JM, Jun ES, Bae YC, Jung JS (2008). Mesenchymal stem cells derived from human adipose tissues favor tumor cell growth in vivo. Stem Cells Dev..

[34] Iyengar P, Combs TP, Shah SJ, Gouon-Evans V, Pollard JW, Albanese C (2003). Adipocyte-secreted factors synergistically promote mammary tumorigenesis through induction of anti-apoptotic transcriptional programs and proto-oncogene stabilization. Oncogene.

[35] Landskroner-Eiger S, Qian B, Muise ES, Nawrocki AR, Berger JP, Fine EJ (2009). Proangiogenic contribution of adiponectin toward mammary tumor growth in vivo. Clin Cancer Res Off J Am Assoc Cancer Res..

[36] Mauro L, Catalano S, Bossi G, Pellegrino M, Barone I, Morales S (2007). Evidences that leptin up-regulates E-cadherin expression in breast cancer: effects on tumor growth and progression. Cancer Res.

[37] Iyengar P, Espina V, Williams TW, Lin Y, Berry D, Jelicks LA (2005). Adipocyte-derived collagen VI affects early mammary tumor progression in vivo, demonstrating a critical interaction in the tumor/stroma microenvironment. J Clin Invest..

[38] Vona-Davis L, Rose DP (2007). Adipokines as endocrine, paracrine, and autocrine factors in breast cancer risk and progression. Endocr Relat Cancer.

[39] Liu H, Kato Y, Erzinger SA, Kiriakova GM, Qian Y, Palmieri D (2012). The role of MMP-1 in breast cancer growth and metastasis to the brain in a xenograft model. BMC Cancer..

[40] Chan BM, Matsuura N, Takada Y, Zetter BR, Hemler ME (1991). In vitro and in vivo consequences of VLA-2 expression on rhabdomyosarcoma cells. Science.

[41] Ho WC, Heinemann C, Hangan D, Uniyal S, Morris VL, Chan BM (1997). Modulation of in vivo migratory function of alpha 2 beta 1 integrin in mouse liver. Mol Biol Cell.

[42] Yang C, Zeisberg M, Lively JC, Nyberg P, Afdhal N, Kalluri R (2003). Integrin alpha1beta1 and alpha2beta1 are the key regulators of hepatocarcinoma cell invasion across the fibrotic matrix microenvironment. Cancer Res.

[43] Yoshimura K, Meckel KF, Laird LS, Chia CY, Park J-J, Olino KL (2009). Integrin alpha2 mediates selective metastasis to the liver. Cancer Res.

[44] Riikonen T, Westermarck J, Koivisto L, Broberg A, Kähäri VM, Heino J (1995). Integrin alpha 2 beta 1 is a positive regulator of collagenase (MMP-1) and collagen alpha 1(I) gene expression. J Biol Chem.

[45] Zutter MM, Santoro SA, Staatz WD, Tsung YL (1995). Re-expression of the alpha 2 beta 1 integrin abrogates the malignant phenotype of breast carcinoma cells. Proc Natl Acad Sci USA.

[46] Zetter BR (1993). Adhesion molecules in tumor metastasis. Semin Cancer Biol.

[47] Madamanchi A, Santoro SA, Zutter MM (2014). α2β1 Integrin. Adv Exp Med Biol.

[48] Kroll SS (2000). Fat necrosis in free transverse rectus abdominis myocutaneous and deep inferior epigastric perforator flaps. Plast Reconstr Surg.

[49] Gao D, Bing C (2011). Macrophage-induced expression and release of matrix metalloproteinase 1 and 3 by human preadipocytes is mediated by IL-1β via activation of MAPK signaling. J Cell Physiol.

[50] Peinado JR, Pardo M, de la Rosa O, Malagón MM (2012). Proteomic characterization of adipose tissue constituents, a necessary step for understanding adipose tissue complexity. Proteomics.

[51] Abe R, Kumagai N, Kimura M, Hirosaki A, Nakamura T (1976). Biological characteristics of breast cancer in obesity. Tohoku J Exp Med.

[52] Calle EE, Rodriguez C, Walker-Thurmond K, Thun MJ (2003). Overweight, obesity, and mortality from cancer in a prospectively studied cohort of U.S. adults. N Engl J Med.

[53] Eliassen AH, Colditz GA, Rosner B, Willett WC, Hankinson SE (2006). Adult weight change and risk of postmenopausal breast cancer. JAMA, J Am Med Assoc.

[54] Ahn J, Schatzkin A, Lacey JV, Albanes D, Ballard-Barbash R, Adams KF (2007). Adiposity, adult weight change, and postmenopausal breast cancer risk. Arch Intern Med.

[55] Cleary MP, Maihle NJ (1997). The role of body mass index in the relative risk of developing premenopausal versus postmenopausal breast cancer. Proc Soc Exp Biol Med Soc Exp Biol Med NY N..

[56] Sestak I, Distler W, Forbes JF, Dowsett M, Howell A, Cuzick J (2010). Effect of body mass index on recurrences in tamoxifen and anastrozole treated women: an exploratory analysis from the ATAC trial. J Clin Oncol Off J Am Soc Clin Oncol..

[57] Carmichael AR (2006). Obesity and prognosis of breast cancer. Obes Rev Off J Int Assoc Study Obes..

[58] Kumar NB, Cantor A, Allen K, Cox CE (2000). Android obesity at diagnosis and breast carcinoma survival: evaluation of the effects of anthropometric variables at diagnosis, including body composition and body fat distribution and weight gain during life span, and survival from breast carcinoma. Cancer.

[59] Borugian MJ, Sheps SB, Kim-Sing C, Olivotto IA, Van Patten C, Dunn BP (2003). Waist-to-hip ratio and breast cancer mortality. Am J Epidemiol.

